# Sore Eyes

**Published:** 1887-03

**Authors:** 


### SORE EYES.

*To the Editor of* THE JOURNAL OF HEALTH :

DEAR SIR — I have been a reader of your magazine for several years, and have gathered much wisdom from its useful pages, and now I feel a greater interest in it than ever before, as I perceive that its articles are less conservative than formerly, and that the views set forth therein are keeping pace with the enlightenment of the age in hiegenic and other matter pertaining to the welfare of the human race. But there is one thing I have never seen in print, which, I fear, has been entirely overlooked by physicians and oculists, and which is the cause of so many people having weak, or slightly diseased eyes, especially of late years.

I attribute the cause, primarily, to this fact : a great many people now-a-days, read newspapers fresh from the press, without thinking, or knowing, that the ink used upon them contains a quantity of arsenic. "Well," the reader may say, "what has this to do with sore eyes ?" Very much, I reply. By handling freshly-printed sheets, very minute quantities of the ink offsets upon the end of the fingers : perhaps, not enough to soil them, but enough to deposit the arsenic thereon. Then, if there is a particle of itching of the eyelids, the persons so affected, almost involuntarily, rub their eyes with the tips of their fingers; and, as the arsenic is a poison, the result is, disease of the eyes ensues, more or less.

Now my advice is, that people wash their hands after handling such newspapers, or, if not convenient to do so, then keep their fingers carefully away from their eyes.

If this advice is strictly adhered to, there would be less use for the physician or the oculist in this particular. It is an old and true saying, that "an ounce of preventive is worth a pound of cure," and if this brief note of caution meets with your views, Mr. Editor, give your readers the benefit of it. AN OLD PRINTER.

---

MIND READING —We have been having a good deal of experimental insight into Mr. Bishop's methods of mind reading during the past week. Prof. Carpenter is still holding his mesmeric seances at the Grand Opera House. We shall have something to say upon the methods of these gentlemen in good time.

